# Childhood diabetes: a myth or reality?- perception of the public from a low-income country: a cross-sectional study

**DOI:** 10.1186/s12889-018-5744-7

**Published:** 2018-07-09

**Authors:** Ugo Nnenna Chikani, Adaobi Ijeoma Bisi-Onyemaechi, Tagbo Oguonu, Shalewa Modupe Ugege, Chinwe Ogugua

**Affiliations:** 1 University of Nigeria Nsukka, Ituku Ozalla campus, Enugu, Nigeria; 2 Department of Paediatrics, UNTH, PMB 01129, Enugu, Enugu State Postal Code 400001 Nigeria; 3 Usman Danfodio University, Sokoto, Nigeria; 4 Federal Teaching Hospital Abakaliki, Abakaliki, Nigeria; 5 Ebonyi State University Abakaliki, Abakaliki, Nigeria

**Keywords:** Childhood diabetes, Public perception, KAP

## Abstract

**Background:**

The diagnosis of Type 1 diabetes is commonly missed in most health facilities in Nigeria. Adequate knowledge of childhood diabetes is necessary for the recognition and possible intervention for the control of the disease. However, research to assess knowledge deficiencies and their relationship to attitude is lacking in most developing countries including Nigeria. This study intends to survey the beliefs and perceptions of caregivers of children towards diabetes in childhood. It also aims at determining the caregivers’ depth of knowledge of diabetes in children, the relationship between knowledge and practices as well as the association between level of education and gender with practices and knowledge. The study outcome will help in the formulation of policy and education of the communities with regards to the preconceived myths and realities of childhood diabetes.

**Methods:**

A descriptive study involving 500 respondents, population groups were selected by multi-staged sampling from different areas in Enugu metropolis, south-East of Nigeria. A validated structured interviewer-administered questionnaire was used. Ethical approval was obtained, and only consenting subjects were interviewed. Data was analyzed with Statistical Package for the Social Sciences (SPSS) version 20.

**Result:**

Almost all the respondents (99.8%) had heard of diabetes in adults. However, a lower proportion of respondents 43.2% knew about diabetes in childhood. Only 24.8% had good knowledge of the different aspects of diabetes. Although females were more knowledgeable about the effects of healthy life style modifications on diabetes, there was no gender predisposition in knowledge about diabetes in childhood.

A positive association existed between knowledge and education [*p* < 0.001] concerning childhood diabetes. Irrespective of this association 82.6% of the respondents with good knowledge of the disease still had a poor attitude towards healthy life style practices.

**Conclusion:**

This study has shown that beliefs and perceptions of childhood diabetes among the adult caregivers in Enugu, south – East Nigeria is mostly erroneous and their knowledge deficient. Literacy did not improve both knowledge and attitude to healthy lifestyle practices.

**Electronic supplementary material:**

The online version of this article (10.1186/s12889-018-5744-7) contains supplementary material, which is available to authorized users.

## Background

Childhood diabetes mellitus is one of the non-communicable diseases diagnosed in children. It is a condition characterized by chronic hyperglycemia resulting from defects in insulin secretion, insulin action, or both [1]. Type 1 diabetes (also referred to as childhood diabetes) accounts for over 90% of childhood diabetes. Diabetes in children is associated with both short and long-term life-threatening conditions which include diabetes ketoacidosis, retinopathy, nephropathy, etc. and these complications are associated with high morbidity and mortality.

The burden of childhood diabetes is on the increase. In 2017, the estimated global population of children (0-14 years) was 1.9 billion, out of which 586,000 children are diagnosed with type 1 diabetes, with estimated 79.000 new cases diagnosed each year [2]. Worldwide, the prevalence among the youths is estimated to triple in 2050 [3]. This projection indicates a serious picture of the future national childhood diabetes burden among the youth and highlights the need to reduce the burden. However, despite this serious scenario, field data [4] suggest that individual country estimates (especially in developing countries) are uncertain or inaccurate. In Nigeria, the frequently cited prevalence of 2.2% in a country of over 180 million people; reported from a community-based study [5] could be an underestimation of the disease compared to the higher prevalence rates reported from some hospital-based studies in Nigeria [6–9] and some high and middle-income countries [3, 10]. The discrepancy could be attributed to lack of awareness of the disease in the community. Within communities there are erroneous beliefs even among health professionals that diabetes occur only in adults, this misconception has led to deaths from unrecognized or misdiagnosed cases [4]. Deaths due to diabetes can be avoided by early recognition of affected children and helping them to survive with appropriate treatment. This will enable them to live a full life void of stigmatization and without restrictions or disabling complications [11]. Early recognition can be achieved through sensitization and creation of awareness. Knowledge about childhood diabetes is a prerequisite for individuals and communities to recognize, report and act upon to control the disease. For one cannot report nor seek attention for what one has no knowledge about. There are few reports on knowledge and attitude on diabetes in developing countries including Nigeria [12]. The most cited reports are those on adult diabetes. However, research to assess knowledge deficiencies and their relation to attitude is lacking in most developing countries including Nigeria.

This study seeks to survey community in Enugu south-East of Nigeria to ascertain the beliefs and perceptions of the public with regards to childhood diabetes. It also aims at determining their depth of knowledge about childhood diabetes and the relationship between knowledge and practices as well as the association between level of education and gender with practices and knowledge. The study outcome will help in the formulation of policy and education of the communities with regards to the conceived myths and realities of childhood diabetes.

## Methods

### Site and population

The study was carried out in Enugu metropolis. Enugu is the capital city of Enugu State in South-East, Nigeria, located at 6° 27′ 9.6“ N, 7° 30’ 37.2” [13]. It is largely populated by members of Igbo ethnic group. The city has hilly geography with a population of 722, 664 [13]. It has a tropical savannah climate with a mean daily temperature of 26.7 °C and average annual rainfall of 2000 mm.

### Study design, subject selection, and ethical considerations

A descriptive, cross-sectional study design whereby population groups in the city were selected by a multi-staged sampling. Enugu metropolis is made up of three local government areas (LGA). The three LGA are made up of 39 wards. Five wards were selected from each of the three LGA by simple random selection using the table of random numbers. The last stage was a consecutive sampling of the individuals in selected households within the selected wards. Consenting subjects who met the inclusion criteria (subject ≥15 years) were selected until the desired sample size was reached. Known diabetics were excluded from the study. Ethical approval was obtained from the Health Research and Ethics Committee of University of Nigeria Teaching Hospital Enugu Ituku-Ozalla (NHREC/05/01/2008B).

### Questionnaire design

A structured interviewer-administered questionnaire was validated by the researchers after a pilot study of 30 respondents was done in Agwu LGA different from the study areas. This was done to assess the suitability of the contents, clarity, sequence, and flow of the questionnaire. The questionnaire was then refined for final use {refer to Additional file 1}.

The first section of the questionnaire covered the respondents’ demographic information which included: age, sex, level of education, occupation, religion, tribe, address, and phone number.

The second section of the questionnaire covered knowledge about diabetes which included knowledge of occurrence/existence of diabetes in children and on different aspects of diabetes. The occurrence of diabetes in children was assessed with the following questions: Have you ever heard of diabetes? Do you think it is communicable? Can it occur in children and what comes to your mind when diabetes is mentioned? Questions on different aspects of diabetes included knowledge on signs and symptoms, causes, complications, and treatment. Respondents were expected to indicate signs and symptoms of diabetes they were familiar with out of 15 options given; cough, chest pain, frequent urination, excessive thirst or hunger, weight loss, coma, stomachache, diahorrea, tiredness, fever, headache, rash, stomach swelling, leg swelling, vomiting. Knowledge on causes was assessed by these options whether there were known diabetes in the family or inherited from the parents, whether it was acquired from eating sugary food/drinks or transmitted from use of toilet, sharing the same cup and plate from a sufferer, lack of insulin or lack of body’s response to insulin and if one can get diabetes by contact with someone that has diabetes or no idea. Knowledge about complications was also requested for. Seven options were given, which included leg ulcer, kidney problem, heart failure, eye problem, poor school performance, stroke and intellectual disability. Respondents’ knowledge of treatment was assessed by these questions, if childhood diabetes was curable or not, the use of insulin and the cost implication. Respondents’ knowledge of diabetes were categorized as either good, little or none depending on their responses: good knowledge represented more than one correct answer while little knowledge was for one correct answer and no knowledge for no correct answer.

The third section of the questionnaire assessed the participants’ attitude towards the treatment of childhood diabetes and healthy lifestyle characteristics such as diet, physical activity, weight monitoring, regular use of insulin, home blood sugar records, abstinence from sugar-rich drinks, hypoglycemic agents, regular malaria treatment and general health-seeking behavior. The health seeking practice was assessed by asking participants to indicate their willingness to adopt those healthy life style modifications and to indicate their roles in the management of childhood diabetes either in prevention, treatment, cure, complication or otherwise.

For this study, knowledge less than 40% was interpreted as poor, 40–70% fair and good for greater than 70%.

## Results

### Subject characteristics

Five hundred individuals participated in the study. More of the respondents (40%, 200/500) were between the age range of 25-34 years with a mean age of 39.3 years. The sex distribution was almost equal; male 50.4%. Respondents were predominantly of the Igbo tribe (99.8%). All were Christians with different educational background: the majority of the respondents had tertiary education (61.6%) while 38.4% had secondary education and below.

### Knowledge of childhood diabetes

Almost all the respondents (99.8%) have heard of diabetes in adults but fewer participants 43.4% were aware of childhood diabetes. Among all the participants 26.2% had good knowledge of the common symptoms of diabetes (Table 1). There were variable proportions of respondents who associated diabetes with excessive thirst/hunger, tiredness and weight loss (Table 2). Diabetic ketoacidosis was only known to a minority (Table 2). Regarding the pathogenesis of diabetes fewer than half of the respondents were knowledgeable of the relationship of insulin to diabetes (Table 3). The complications of diabetes were equally known to a few of the participants (Table 4). Treatment of childhood diabetes with  life long insulin were known to fewer than half of the respondents (Table 5).

**Table 1:** Summary of levels of knowledge on different aspects of diabetes

Knowledge of diabetes	Symptoms and signs	Causes	Treatment	Complications
Good knowledge (more than one correct answer)	131 (26.2)	87 (17.4)	233 (46.6	207 (41.4)
Little (one correct answer)	295 (59.0)	–	92 (18.4)	204 (40.8)
None	74 (14.8)	413 (82.6)	175 (35.0)	89 (17.8)
Total	500 (100)	500 (100)	500 (100.0)	500 (100)

**Table 2:** Responses to specific questions on knowledge of different aspects of diabetes

Signs and symptoms	Yes (%)	No (%)
Cough	11 (2.2)	489 (97.8)
Chest pain	95 (19)	405 (81.0)
Frequent urination	426 (85.2)	74 (14.8)
Excessive thirst or hunger	96 (19.2)	404 (80.8)
Stomach ache	31 (6.2)	496 (93.8)
Tiredness	115 (23)	385 (77)
Rash	3 (0.6)	497 (99.4)
Leg swelling	151 (30.2)	349 (69.8)
Stomach swelling	37 (7.4)	463 (92.6)
Vomiting	28 (5.2)	472 (94.4)
Headache	56 (11.2)	444 (88.8)
Diahorrea	36 (7.2)	464 (92.8)
Coma	11 (2.2)	489 (97.8)
Weight loss	75 (15)	425 (85)
Fever	73 (14.6)	427 (85.4)

**Table 3:** Responses to specific questions on knowledge of Causes

Causes	Yes Number (%)	NO Number (%)
It runs in the family/ Inherited from the parents	322 (64.4)	178 (35.6)
Acquired from eating sugary things/drinks	370 (74.0)	130 (26.0)
Transmitted from the toilet	3 (0.6)	497 (99.4)
Sharing the same cup and plate from a sufferer	6 (1.2)	494 (98.8)
Contact with someone that has the sickness.	2 (0.4)	498 (99.6)
Complete lack of insulin	31 (6.2)	469 (93.8)

**Table 4:** Responses to questions on knowledge of complications: The following are complications associated with childhood diabetes

Complications	Yes (frequency %)	No (frequency %)
Leg ulcer	289 (57.8)	211 (42.2)
Kidney problem	232 (46.4)	268 (53.6)
Heart failure	70 (14.0)	430 (86.0)
Eye problem	86 (17.2)	414 (82.8)
Poor school performance	50 (10)	450 (90)
Stroke	82 (16.4)	418 (83.6)
Intellectual disability	2 (4)	498 (99.6)

**Table 5:** Responses to specific questions on knowledge of treatment

Questions	Yes frequency %	No frequency %
Is it treatable	443 (88.6)	57 (11.4)
If yes how medical?	403 (80.6)	97 (19.4)
Herbal medications	49 (9.8)	451 (90.2)
Diet	223 (44.6)	277 (55.4)
Spiritual	478 (22)	95.6 (4.4)
Use of insulin for life ie daily injection of insulin	187 (37.4)	313 (62.6)
Lifelong hospital follow up	172 (34.4)	328 (65.6)
Oral hypoglycemic drugs	308 (61.6)	192 (38.4)
Bitter leaf water	42 (82)	458 (91.6)
Multivitamin	11 (22)	489 (97.8)
Intravenous infusion	5 (1)	495 (99)
Paracetamol	8 (1.6)	492 (98.4)
Surgery	5 (1)	495 (99)
Genetics	165 (33)	335 (67)
Curse	11 (2.2)	489 (97.8)
Can one achieve a total cure	369 (73.8)	131 (26.2)

Knowledge of features of diabetes in children did not confer appropriate health seeking behaviors to the respondents (*p* value< 0.001) (Table 6).

**Table 6:** Relationship between knowledge and attitude and practice

Assessment	Knowledge	Attitude and practice		‘Total
		Good	Poor	
Good	Cause	5	82	87
Little or None		81	332	413
Good	Signs and Symptoms	22	109	131
Little or None		64	305	368
Good	Complications	58	149	207
Little or None		28	265	292
Good	Treatment	41	135	186
Little or None		47	279	264

### Association between knowledge of diabetes and the social characteristics of the respondents

There is a statistically significant association between the respondents’ level of knowledge and their educational attainment (*p* < 0.001)**.** Although there was no gender difference regarding the knowledge of childhood diabetes, the attitude of females towards the life style modification in treatment of diabetes was better (*p* < 0.06).

## Discussion

The study outcome has shown a fair knowledge of childhood diabetes among the adult population in Enugu metropolis. In contrast, the majority of these respondents (99.8%) were aware of diabetes in adults consistent with findings from previous studies on knowledge of diabetes in adults [12, 14–19]. The knowledge level of respondents in this study could be attributed to the fact that majority were educated and most likely have inferred that since diabetes occurs in adults it is also likely to occur in children. Despite this knowledge base the individuals interviewed showed an inability to recognize the etiology and features of the disease.

Diabetes in children is caused by lack of insulin, hence lifelong daily insulin therapy is an integral component among other treatment modalities in the management of childhood diabetes. The knowledge was lacking among the participants and thus poses a significant obstacle to treatment and prevention of complications in the childhood disease. Strangely enough the consumption of sugary substances seems to be the dominant held belief. For instance, a majority recommended the use of oral hypoglycemic agents as a treatment option for childhood diabetes, which is probably an inference from the treatment of diabetes in adults. The deficiency in knowledge of the cause, as well as treatment calls for urgent intensive education of the public on childhood diabetes with emphasis on the fact that childhood diabetes is not a smaller version of diabetes in adult but a distinctive pathology in children. Mass education should be done by all concerned; diabetes and health educators, social worker, doctors and other health care providers through the use of information, education and communication systems to disseminate the appropriate facts.

Irrespective of the knowledge level of the presence of the disease, the ability to recognize the common symptoms and possible complications is paramount in health seeking behavior as well responding to advice from health care professionals. The paucity of knowledge shown by the respondents of the features of childhood diabetes implies that there is a considerable risk that the disease state may not be recognized early and complications averted. Of particular note is the non-recognition of diabetes ketoacidosis as a life-threatening condition of childhood diabetes. This is worrisome considering that 90% of children with diabetes will present in DKA (medical emergency) before the first diagnosis [1].

Long-term complications are usually associated with exposure of organs to chronic hyperglycemia in poorly managed or unrecognized cases. The complications are usually evident from adolescent period into adulthood. The respondents demonstrated good knowledge of diabetes complications compared to the other aspects of childhood diabetes. Not surprising is the recognition as a complication the foot and kidney problems which is presumably an extrapolation from their knowledge of complications of adult diabetes. The disparity in knowledge of features and complications of childhood diabetes was consistently reported in other studies [12, 14, 15, 20].

Female respondents are more likely to seek care than their male counterparts an attribute which should be leveraged upon in line with the World Health Organization/United Nations International Children’s Emergency Fund (WHO/UNICEF) child survival strategies which encourages the role of women in finding health solutions in the communities.

The contrasting finding of poor attitude to appropriate health practice and good knowledge of childhood diabetes demonstrates a poor application of knowledge which will engender a poor outcome of the disease. Similar findings have been reported by Adibe et al. [12] in Nigeria, Wee and colleagues [14] in Singapore demonstrating probably a non-cultural tendency towards childhood diabetes. The observed poor attitude to healthy lifestyle practices was also documented by Guatam et al. in Nepal [15], which was attributed to overconfident and burn out syndrome. However, their study included those with and without diabetes. Failure to recognize the role of insulin and unwillingness allow for long term usage in their wards as well as the respondents reluctance to adopt healthy life style modifications compound the treatment of the children with diabetes and foster poor outcome.

## Conclusion

This study has shown that beliefs and perceptions of childhood diabetes among the adult caregivers in Enugu, south-East Nigeria is mostly erroneous and their knowledge deficient. Literacy did not improve both knowledge and attitude to healthy lifestyle practices. Correction of the attitude and practice may play a substantial role in the recognition and management of childhood diabetes.

## Additional file

Additional file 1: Blank questionnaire on childhood diabetes: a myth or reality?- Perception of the public from a low-income country. (DOCX 17 kb)

## Abbreviations

KAP: Knowledge, attitude and practice
LGA: Local government areas
NHREC: Nsukka health research and ethics committee
SPSS: Statistical package for the social sciences
UNICEF: United Nations International Children’s Emergency Fund
WHO: World Health Organization
