# Supplementary material for: Childhood diabetes: a myth or reality?- perception of the public from a low-income country: a cross-sectional study
Source: BMC Public Health. 2018 Jul 9;18:852. doi: 10.1186/s12889-018-5744-7 (PMC6038320; doi:10.1186/s12889-018-5744-7)
Supplement: Supplementary file 1 — Blank questionnaire on childhood diabetes: a myth or reality?- Perception of the public from a low-income country. (DOCX 17 kb) [file 12889_2018_5744_MOESM1_ESM.docx]

ADDITIONAL FILE

File name: Additional file 1

Title of Data: Blank Questionnaire on **Childhood diabetes: a myth or reality?- Perception of the public from a low-income country .**

**Biodata: Section A: Circle as appropriate**

**Age:…………** (15-24 years) (25-34 years) (35- 44 years) (45-54 years) 55 years and above

**Sex:** M/F, **phone no-------------------------, Address/email----------------------------**

**Occupation---------------------Tribe---------------Religion------------------------------------- place of interview _____________________**

**Highest Educational status:** None, primary, secondary, tertiary, vocational

**Section B**

1. **Knowledge**
2. **Levels of community perception/awareness on occurrence of diabetes in children:**
3. Have you heard of diabetes? a)Yes b) No
4. What comes to your mind when diabetes is mentioned?

a) physical illness b) spiritual c)curse/punishment d) myth d)others………………………………………

1. Can diabetes occur in children? a) yes b)no C) don’t know
2. Do you think it is communicable? a) yes b) no c)don’t know
3. **Levels of community knowledge on different aspects of diabetes:**
4. **Signs and symptoms:** A child with diabetes will have the following:

(a)cough(b) chest pain (c) frequent urination (d) excessive thirst or hunger (e) weight loss (f) coma (g) tommy ache (h) diahorrea (i)tiredness( j)fever, (k)headache (l) rash (m) tommy swelling (n)leg swelling (o) vomiting

**B) Causes: mode of inheritance-How does one get diabetes?**

(a) It runs in the family/ Inherited from the parents b) acquired from eating sugary things/drinks (c) transmitted from the toilet (d)sharing the same cup and plate from a sufferer (e) lack of insulin(f)contact with someone that has the sickness.(g)I don’t know.

**C)Complications:**(a) leg ulcer (b) kidney problem (c) heart failure (d)eyeproblem (e) poor school performance (f) stroke ( g) intellectual disability

**D) Treatment:**

Is it treatable?- a)Yesb)No

I**f yes –how?** (a) Medical (b) Native(c) Spiritual (d) diet

**If medical**

**Which of the following are used?**

- 1. Use of insulin for life ie daily injection of insulin (b)lifelong hospital follow up

(c)Diabetic drugs (d) bitter leaf water (e) Drip (f)multivitamins (g) paracetamol (h) surgery (i)others(specify)

**Curable:** Can you achieve total cure? (a)Yes (b)No

**If No, why?**-(a ) genetic (b) lifelong disease (c) curse

**What is the cost implication?** (a)Expensive (b)moderate (c) cheap (d) no idea

1. **ATTITUDE**

**Community (public) attitude and practices toward diabetes**

Assessment of the attitude of community towards lifestyle characteristics: (a)Good (b) poor (c) unwilling

Which of the following has a role in treatment of diabetes in children? (**you can tick more than one)**

1. Diet,
2. Physical activity( regular exercise)
3. Health seeking behaviour
4. Weight monitoring
5. Regular use of insulin
6. Home blood sugar records
7. Abstinence from sugar rich drink and snacks
8. Diabetic drugs
9. Treating for malaria regularly

Explain the roles of the options selected above

1. Diet--- a)prevention b)treatment ( c) cure (d) complication (e)others(specify)
2. Physical activity( regular exercise) -a)prevention b)treatment ( c) cure (d) complication (e)others(specify)
3. Health seeking behavior-a)prevention b)treatment (c) cure (d) complication (e)others(specify)
4. Weight monitoring-a) prevention b) treatment ( c) cure (d) complication (e)others(specify)
5. Regular use of insulin-a) prevention b) treatment (c) cure (d) complication (e)others(specify)
6. Home blood sugar records-a) prevention b) treatment (c) cure (d) complication (e)others(specify)
7. Abstinence from sugar rich drink and snacks -a) prevention b) treatment (c) cure (d) complication (e)others(specify)
